# Scaling of Avian Primary Feather Length

**DOI:** 10.1371/journal.pone.0015665

**Published:** 2011-02-09

**Authors:** Robert L. Nudds, Gary W. Kaiser, Gareth J. Dyke

**Affiliations:** 1 Faculty of Life Sciences, University of Manchester, Manchester, United Kingdom; 2 Royal British Columbia Museum, Victoria, Canada; 3 School of Biology and Environmental Science, University College Dublin, Dublin, Ireland; Raymond M. Alf Museum of Paleontology, United States of America

## Abstract

The evolution of the avian wing has long fascinated biologists, yet almost no work includes the length of primary feathers in consideration of overall wing length variation. Here we show that the length of the longest primary feather (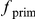) contributing to overall wing length scales with negative allometry against total arm (*ta* = humerus+ulna+manus). The scaling exponent varied slightly, although not significantly so, depending on whether a species level analysis was used or phylogeny was controlled for using independent contrasts: 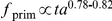. The scaling exponent was not significantly different from that predicted (0.86) by earlier work. It appears that there is a general trend for the primary feathers of birds to contribute proportionally less, and *ta* proportionally more, to overall wingspan as this dimension increases. Wingspan in birds is constrained close to mass (*M* ^1/3^) because of optimisation for lift production, which limits opportunities for exterior morphological change. Within the wing, variations in underlying bone and feather lengths nevertheless may, in altering the joint positions, permit a range of different flight styles by facilitating variation in upstroke kinematics.

## Introduction

**Figure 1:**
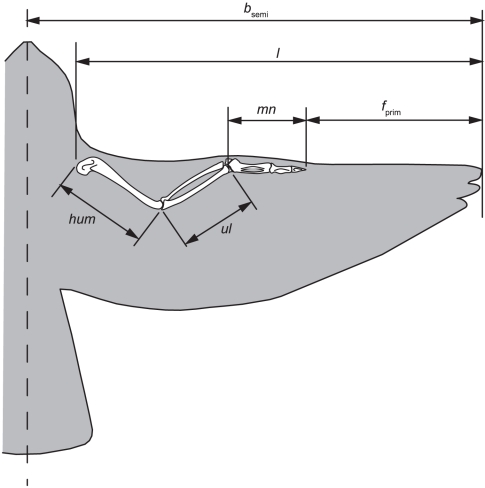
Diagram showing the measurements taken from the museum specimens and used in the analyses (adapted from **figure 1** in [1]).

The total length of the avian wing derives from the underlying wing bones (humerus, radius/ulna and manus) and the functional primary feathers (Fig. 1). Although scaling exponents vary slightly depending upon whether the effects of common ancestry are controlled for using independent contrasts or not (*M* ^0.35^ and *M* ^0.39^ respectively, table 1 in [1]), it is well established that wingspan (*b*) in birds scales with slightly positive allometry with respect to body mass (*M* ^>1/3^) [1]–[4]. This positive allometry, however, appears related to size dependent variation in flight behaviour [1]. Specifically, the line of best fit is depressed at lower body masses and elevated at high body masses, because slow speed flapping flight styles seen in smaller birds are associated with short-wings, while the soaring flight styles of larger birds favour longer wings [4]. Surprisingly, and in spite of variations in flight behavior, the relative contribution of the primary feathers to overall wing length has received little attention from ornithologists.

**Table 1:** The primary feather and total arm data set.

Number in figure 2a	Species	*n*	Total-arm (m)	Primary feather (m)
1	*Aegolius acadicus*	1	0.131	0.108
2	*Anas americana*	2	0.223	0.200
3	*Anas crecca*	1	0.150	0.165
4	*Anas platyrhynchos*	1	0.245	0.233
5	*Anthus spinoletta*	2	0.066	0.066
6	*Ardea herodias*	1	0.611	0.378
7	*Aythya marila*	1	0.225	0.159
8	*Bombycilla garrulus*	2	0.081	0.086
9	*Bubulcus ibis*	4	0.252	0.182
10	*Butorides striatus*	1	0.205	0.160
11	*Cathartes aura*	1	0.465	0.428
12	*Ceryle alcyon*	10	0.147	0.139
13	*Dendragapus obscurus*	3	0.190	0.184
14	*Dendroica coronata*	2	0.053	0.054
15	*Dendroica magnolia*	1	0.045	0.044
16	*Egretta thula*	1	0.245	0.187
17	*Eremophila alpestris*	2	0.079	0.084
18	*Gavia adamsii*	1	0.518	0.252
19	*Gavia immer*	2	0.510	0.217
20	*Glaucidium gnoma*	1	0.091	0.070
21	*Mniotilta varia*	2	0.051	0.058
22	*Oceanodroma furcata*	4	0.108	0.120
23	*Parus hudsonicus*	2	0.045	0.054
24	*Passerculus sandwichensis*	2	0.064	0.061
25	*Phalacrocorax pelagicus*	1	0.308	0.207
26	*Pheuctitis ludovicianus*	2	0.077	0.078
27	*Pipilio erythrophthalmus*	2	0.066	0.069
28	*Piranga ludovicianus*	2	0.068	0.072
29	*Puffinus griseus*	14	0.298	0.194
30	*Puffinus tenurostris*	1	0.248	0.189
31	*Seiurus aurocapillus*	2	0.058	0.058
32	*Setophaga ruticilla*	2	0.045	0.050
33	*Sitta canadensis*	2	0.050	0.049
34	*Sphyrapicus ruber*	3	0.091	0.107

Curiously, total-arm (*ta* = humerus+ulna+manus) length does not scale with unity against wing semi-span *b*_semi_ and instead appears to scale with positive allometry (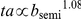), indicating that larger birds have longer wings relative to their *M*, but also have longer *ta* relative to their *b* [1]. An explanation forwarded to explain this disproportionate increase in *ta* with *b* [1] was that primary feather length (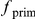) is relatively shorter in longer winged birds (i.e., 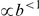). The only data available, however, suggested 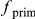 scaled as *M* ^0.32^, which was not significantly different from the exponent predicted for isometry (*M* ^1/3^) [5]. Worcester's [5] study was, however, limited in taxa (*n* = 13) and, because the relationship between *b* and *ta* was not determined, whether increasing *M* in the sample correlated with a relatively longer *ta* was not known. Therefore, a trend towards shorter primaries in birds with longer *ta* remains a possibility [1] and required further investigation. Nudds [1] also acknowledged that if elbow angle varied with *b* it would influence how close to parallel the leading edge of the humerus and ulna was and hence the relationship between *ta* and *b* (Fig. 1). Elbow angle is extremely difficult to measure in live birds, however, because bones are not visible from the wing-surface. Plucking of feathers is unethical and undesirable, and x-ray not necessarily practical, but if negative allometry was found between 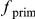 and *ta* then the effect of elbow angle could be ruled out.

‘Stretched’ or ‘flat’ wing preservations are rare in museum collections and those including the humerus intact within the skin are even more so (personal observations). However, a small collection of suitably stretched wing specimens was located in the Royal British Columbia Museum, Victoria, BC, Canada (RBCM). Even these had the humerus removed from the wing, but fortunately kept separately to permit all wing-bone measurements to be recorded from a homogenous specimen. These skins allowed us to test the hypothesis that 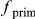 scales with negative allometry against *ta* (i.e., 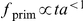) as proposed by Nudds [1]. More specifically it was predicted that, over the range of wing semi-spans (*b*_semi_ = 0.075 to 1.622 m) used in Nudds [1], the predicted scaling exponent between 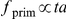 should approximate to 0.86, because 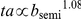 and, 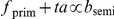, so 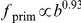 and therefore 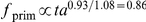. A predicted exponent of 0.86 assumes that size dependent variation in 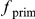 is entirely responsible for the positive allometry seen in *ta* (i.e., elbow angle is constant across all wingspans).

## Methods

Humerus, ulna and manus lengths were measured using Vernier calipers to the nearest mm from the ‘spread wing’ bird skin collection at the RBCM. ‘Total-arm’ is the sum of humerus, ulna and manus length [3], [6]. Primary feather length (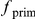) was measured from the distal end of digit 2 of the manus to the feather tip, parallel to the feather shaft (Fig. 1). The primary feather chosen was that contributing the most distal point of the wing representing maximum *b*.

Because the data set comprises interspecific measures (Table 1), the effects of common ancestry must be considered to prevent spurious correlations resulting from common descent rather than from independent evolution. Here a comparative analysis using standardized independent contrasts, conducted in CAIC version 2.6.9 [7], was used. The analyses were implemented in three ways. Initially the scaling relationships were calculated using species as independent data points. The analysis was then repeated using CAIC and the phylogenetic hypotheses of Sibley and Ahlquist [8], and finally CAIC was implemented using the phylogenetic hypotheses of Livezey and Zusi [9]. A punctuated model of evolution was used in both cases: the branch length estimates of Sibley and Ahlquist [8] are disputed and none are available for the phylogeny of Livezey and Zusi [9]. The topological disagreement between these two hypotheses [8], [9] is useful, because if phylogeny is going to affect the results, then using two different phylogenies is likely to have a greater effect than changes to branch lengths within a single phylogeny. Use of two different phylogenies should therefore indicate whether the scaling relationships determined are likely to be affected by future refinements of phylogenetic topology.

The relationship between 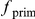 and *ta* was investigated using the empirical scaling formula 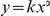 where *α* is the allometric exponent (slope) and *k* is the allometric coefficient (intercept), which was in turn determined using a Model II reduced major axis (RMA) regression [10]–[12]. Regression analyses using independent contrasts were performed through the origin [7]. The RMA slope was calculated as the ordinary least squares (OLS) Model I slope (regression coefficient) divided by the OLS correlation coefficient, and 95% confidence limits were calculated following Sokal and Rohlf [13]. The standard error (s.e.) of the RMA slope was taken as equal to that of the s.e. of the OLS slope. Two-tailed *t*-tests were used to test for differences between calculated slopes and the slopes predicted for geometric similarity (*α* = 1) or predicted from Nudds [1] (*α* = 0.86).

## Results

**Figure 2:**
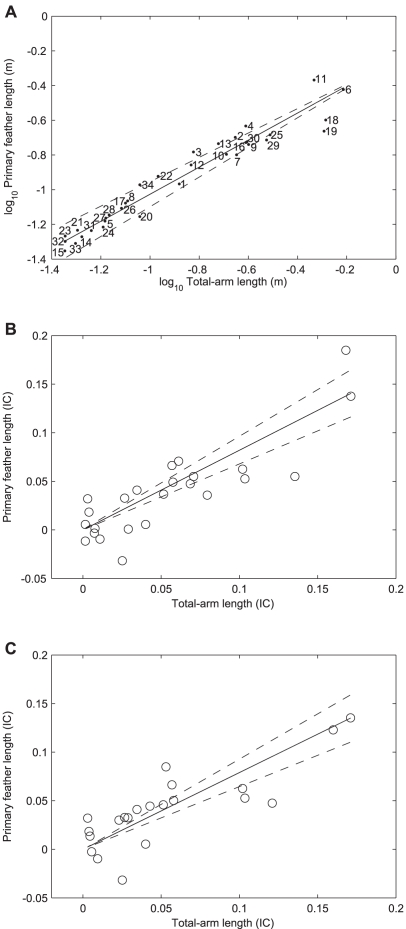
Scatter plots of log_10_ primary feather length (m) against log_10_ total-arm length (sum of humerus, ulna and manus length in m). The regression lines (dashed lines are 95% C.I.s) describing the relationship were A) species treated as independent data points: *y* = 0.57*x* ^0.78 (0.71–0.85)^, *t* = 23.35, *n* = 34, *r* ^2^ = 0.94, *p*<0.001, B) phylogenetic independent contrasts (IC) using the phylogeny of Livezey and Zusi [9]: *y* = *x* ^0.82 (0.68–0.96)^, *t* = 12.26, *n* = 24, *r* ^2^ = 0.84, *p*<0.001 and C) phylogenetic independent contrasts (IC) using the phylogeny of Sibley and Ahlquist [8]: *y* = *x* ^0.79 (0.64–0.93)^, *t* = 11.46, *n* = 21, *r* ^2^ = 0.84, *p*<0.001. See table 1 for the species corresponding to the numbers in panel A.

In all three analyses the relationship between 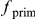 and *ta* was statistically significant, with the scaling exponent dependent upon the analysis used (Fig. 2). The scaling exponent determined using species as independent data points was significantly below (*t* = −6.50, *p*<0.001) that predicted for geometric similarity (*α* = 1). Similarly, both CAIC using the phylogeny of Livezey and Zusi [9] and the phylogeny of Sibley and Ahlquist [8] produced slopes significantly below 1 (*t* = −2.72, *p*<0.05 and *t* = −3.12, *p*<0.05 respectively). In all three cases the scaling exponents were below, yet not significantly different from, that predicted (i.e., 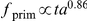) by Nudds [1] as demonstrated by the 95% confidence intervals (Fig. 2).

## Discussion

As predicted by Nudds [1] and contrary to that suggested by the data of Worcester [5], there is a general trend for the primary feathers of birds to contribute proportionally less to overall wing-length as *b*_semi_ increases. The sample size here was relatively small (*n* = 34) compared to the sample sizes (*n* = 306) used to investigate the scaling of *ta* [1], which precludes any analysis of flight style or ecologically driven variation in 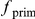/*ta* ratio. The wingspan of birds is constrained close to *M* ^1/3^, because of optimisation for lift, limiting the opportunities for exterior morphological change. Within the wing, however, variations in underlying bone ratios may permit a range of different flight styles, by possibly facilitating variation in upstroke kinematics [6]. It is not unreasonable to expect the relationship between 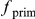 and *ta* to also vary depending upon the ecology or flight style of the bird.

The scaling relationship determined here between 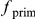 and *ta* does not entirely exclude the possibility of size dependent variation in elbow angle. Although there were no statistical differences between the calculated scaling exponents (Fig. 2) and the 0.86 predicted [1], they were lower (0.78–0.82) and the 95% confidence intervals broad. Of course, the angle at the elbow in a stretched out wing when a bird is having its wingspan measured [14] is not necessarily functional. Instead, it could just be an artefact of how the bird is held by the researcher. Indeed, the elbow angle is likely varied in flight and during a wing-stroke [15], [16]. This, of course, begs the question of exactly what we measure when we measure *b* in a bird: it may be maximum extended wingspan, but is this used during flight? In hummingbirds, span in flight is effectively the width of the body plus the distances from the wrists to wing tips [17], but in other birds the portions of the wings between the wrists and the body need to be considered [18]. Measurements of functional wingspan from birds in flight are long overdue.

In conclusion, there is a general trend for 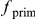 to contribute relatively less to overall wingspan in larger birds. Conversely, *ta* contributes more to the overall length with increasing *b*. Why this trend exists is not immediately obvious. Although tentative at this stage, the scaling of *ta* and 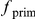 may be the product of an as yet unidentified optimum ratio for feathers to wing-skeleton length within the biomechanical and aerodynamic constraints acting upon the scaling of *b* (*M* ^1/3^) [1]. Similarly, whether the scaling is driven by aerodynamics, feather biomechanical properties or a combination of both requires further investigation.
